# Eating Disorder Symptomatology and Identity Formation in Adolescence: A Cross-Lagged Longitudinal Approach

**DOI:** 10.3389/fpsyg.2018.00816

**Published:** 2018-06-04

**Authors:** Margaux Verschueren, Laurence Claes, Annabel Bogaerts, Nina Palmeroni, Amarendra Gandhi, Philip Moons, Koen Luyckx

**Affiliations:** ^1^Faculty of Psychology and Educational Sciences, KU Leuven, Leuven, Belgium; ^2^Faculty of Medicine and Health Sciences, Collaborative Antwerp Psychiatric Research Institute, University of Antwerp, Antwerp, Belgium; ^3^Department of Public Health and Primary Care, KU Leuven, Leuven, Belgium; ^4^Institute of Health and Care Sciences, University of Gothenburg, Gothenburg, Sweden

**Keywords:** eating disorder symptomatology, identity synthesis, identity confusion, adolescence, cross-lagged, body dissatisfaction, drive for thinness, bulimia

## Abstract

**Introduction:** Eating disorder symptomatology, comprising both psychological and behavioral aspects of subclinical eating concerns, constitutes a clear precursor of developing eating disorders. It is crucial to investigate its antecedents and correlates to subsequently inform eating disorder prevention programs. The present study focused on identity formation, a core developmental task in adolescence, that has increasingly been linked to eating disorder development. Our main aim was to examine the temporal sequence between eating disorder symptomatology and identity formation.

**Methods:** Data on eating disorder symptomatology and identity formation were collected in 530 high school students (at Time 1: mean age = 15 years; *SD* = 1.84; range: 12–18 years; 50.6% females) using self-report questionnaires at three annual measurement points. Cross-lagged structural equation modeling was performed to examine the directionality of effects.

**Results:** Results indicated bidirectional effects between eating disorder symptomatology and identity formation. Identity confusion seemed to increase vulnerability to body dissatisfaction and bulimia symptoms, whereas identity synthesis seemed to protect against their development. Additionally, identity synthesis seemed to protect against the development of drive for thinness as well. At the same time, body dissatisfaction and bulimia symptoms positively predicted identity confusion and negatively predicted identity synthesis over time.

**Conclusion:** The present study adds to the growing body of literature on identity and eating disorders by focusing on their temporal interplay in a community sample of adolescents. As bidirectional effects emerged, a greater emphasis on identity formation in eating disorder prevention programs is advocated.

## Introduction

Adolescence is one of the most turbulent and challenging life periods both physically and psychologically. Especially younger adolescents undergo various bodily changes due to the emergence of secondary sex characteristics such as hair growth, (possible) breast development, and shifts in the accumulation and location of body fat (Tanner, 1981; Susman et al., 2010). These bodily changes are new to young teenagers and can give rise to feelings of insecurity about their physical appearance, especially as adolescents are often greatly concerned with social acceptance and how others perceive them (Elkind and Bowen, 1979; Harter, 1999). Moreover, this age period seems to be specifically at risk to internalize the unrealistic western beauty ideals that promote thin and muscular bodies for girls and boys, respectively (Suisman et al., 2014). This internalization refers to both cognitively and behaviorally complying to social ideals of attractiveness (Thompson et al., 1999) and is regarded a key contributor to developing a negative body image and, especially for girls, a wish to be thinner (Thompson and Stice, 2001; Stice, 2002; Vartanian, 2009; Lawler and Nixon, 2011; Westerberg-Jacobson et al., 2012).

Research has demonstrated that approximately 85% of boys and girls are unhappy with some aspects of their body (Ricciardelli and McCabe, 2001), and an alarming number of adolescents (50–60%) and emerging adults (approximately 25%) admit to experiment with disturbed eating behaviors (e.g., fasting, taking diet pills or laxatives, vomiting, binge-eating; Croll et al., 2002; Quick and Byrd-Bredbenner, 2013). Additionally, these maladaptive eating behaviors are associated with several indicators of psychological ill-being such as depressive symptoms, low self-esteem, and stress (Fryer et al., 1997; Franko and Omori, 1999; Liechty and Lee, 2013) and are considered major risk factors for the development of eating disorders (ED; Stice, 2002; Neumark-Sztainer et al., 2006). Therefore, it is crucial to examine potential contributing factors to disturbed eating behaviors in adolescents and emerging adults. Identity formation constitutes a key developmental task in this age period (Erikson, 1963) and has already been linked to disturbed eating behaviors in both community and clinical cross-sectional studies (Bruch, 1981; Schupak-Neuberg and Nemeroff, 1993; Stein and Corte, 2007; Vartanian, 2009; Verschueren et al., 2017a). Moreover, the promotion of a healthy identity development has been advocated in ED prevention programs (Corning and Heibel, 2016). The present longitudinal study aimed to substantially extend this research line by examining the directionality of effects linking identity and ED symptomatology over time.

In his seminal theory on lifespan personality development, Erikson (1968) described the psychosocial task of developing a clear personal identity as being central in adolescence and young adulthood. However, this task can be very challenging as adolescents need to reconcile both personal needs and social expectations (referred to as the identity crisis). Especially in early to mid-adolescence, teenagers still have a lot of uncertainties about who they are or what they find important in life. *Identity confusion* entails the individual’s feeling of being mixed up and lacking a clear sense of purpose and direction in life. Several researchers have already described that a certain degree of identity-related confusion and distress may be normative throughout adolescence and can even be constructive for later identity work (Erikson, 1956; Marcia, 2006; Meeus, 2011). Yet, it has been related to maladaptive psychosocial functioning as well. Individuals scoring high on identity confusion often report more depressive symptoms, anxiety, and lower self-esteem (Crocetti et al., 2011). In contrast to identity confusion, Erikson (1968) proposes a state of *identity synthesis* in which individuals attain a set of self-identified ideals, values, and goals, and experience themselves as an integrated whole. This identity state is regarded a successful resolution of the identity crisis and has been associated with psychological well-being and greater life-satisfaction (Erikson, 1968; Schwartz et al., 2011). Hence, Erikson (1968) refers to identity confusion and synthesis as polar outcomes of the identity continuum.

Focusing on general identity problems, several researchers and theorists have described how difficulties in identity formation can be related to ED symptomatology in both community and clinical samples. Identity confusion has been found to predict an internalization of certain societal standards (e.g., thin ideal), which, in turn, predicts dieting thoughts and behavior in women (Vartanian, 2009). Similarly, individuals that conform to prescriptions and values of significant others when dealing with identity-relevant information (labeled a normative identity style; Berzonsky, 2004) are more likely to adopt the body perfect ideal, which, in turn, predicts appearance-focused eating regulation and rigid dietary restrictions. In addition, avoiding to face identity issues (labeled a diffuse-avoidant identity style; Berzonsky, 2004) predicts a decrease in health-focused eating regulation (Verstuyf et al., 2014). In line with these findings, patients with an ED have been found to experience more identity confusion when compared to individuals without this diagnosis (Schupak-Neuberg and Nemeroff, 1993; Sparks, 1993; Luyckx et al., 2015; Verschueren et al., 2017a). Although these studies have established a close relationship between identity and ED symptomatology, it is unclear how identity synthesis and confusion on one hand and disturbed eating behaviors and cognitions on the other hand predict one another over time.

In general, most theorists assume that identity problems are already present before the onset of ED symptomatology. Bruch (1981) stated that adolescents scoring high on identity confusion are especially vulnerable to adopt a maladaptive search for a clear sense of identity. As body weight is highly controllable and culturally valued, these adolescents may use their body as an improper source of self-definition, with drive for thinness actually representing a quest for individuation (Bruch, 1981; Casper, 1983). Schupak-Neuberg and Nemeroff (1993) also stated that patients with an ED use their physical bodies to represent their inner identity. In this way, regulation of the body – by controlling food intake – would represent a regulation of one’s own identity. In addition to dietary restraint and drive for thinness, binge eating behaviors have been related to identity confusion as well. Moreover, they have been described as a functional mechanism to avoid dealing with identity issues (Herman and Polivy, 1988; Wheeler et al., 2001). Heatherton and Baumeister (1991) described in their escape theory that, when individuals with binge eating fail to meet their high inner standards, they experience negative thoughts and evaluations about themselves, accompanied by emotional distress. As a way to escape such aversive self-awareness, these individuals may adopt cognitive narrowing strategies in which they narrow their attention to the bodily sensations of eating (i.e., binge eating). Binge eating could thus represent a way to escape from engaging in identity work.

In contrast to most theorists, Corning and Heibel (2016) highlight that ED symptomatology may hinder identity development as well, instead of only focusing on the harmful effects that identity confusion may have on eating behaviors. They argue that most individuals with disturbed eating behaviors are preoccupied with their body weight and shape, and, hence, body image may represent a disproportionate aspect of their identity. Alternate sources of self-esteem are lacking, which makes them especially vulnerable when being confronted with weight teasing or social comparison (Corning and Heibel, 2016). These events are then often experienced as direct threats to their sense of identity. As a result, Corning and Heibel (2016) advocate for prevention programs that teach adolescents to build a broader identity by bolstering other self-aspects than body image or appearance, which may, in turn, allow them to experience a normative trajectory of identity development.

The evidence reviewed so far seems to point to a clear association between identity formation and ED symptoms. However, to our knowledge, no study thus far has addressed the directionality of effects linking these variables over time. Similarly, although different theories in the existing literature seem to adhere to the idea of identity problems increasing vulnerability to ED symptoms, there has been little to no empirical investigation of this tenet. Accordingly, the present three-wave longitudinal study investigated the temporal sequence of ED symptomatology and identity formation over a 2-year period in a community sample of adolescents and emerging adults. This approach was aimed at increasing our understanding of how these constructs predict one another over time and, hence, to unravel the directionality of effects in the identity-eating symptomatology interplay. Inspired by earlier studies, we expected that identity confusion would increase vulnerability to ED symptomatology and identity synthesis would protect against the development of these symptoms (Casper, 1983; Wheeler et al., 2001; Kamps and Berman, 2011). However, as more recent theorizing also proposes the opposite directionality of effects (Corning and Heibel, 2016), we hypothesize that ED symptomatology may also hamper identity synthesis and increase vulnerability to identity confusion. In sum, we hypothesize bidirectional associations to occur in the identity – eating behaviors interplay, with identity problems predicting ED symptomatology over time and ED symptomatology hindering normative identity development as well.

The present study focuses on both psychological and behavioral ED symptoms (body dissatisfaction, drive for thinness, and bulimia symptoms), which have been described as central features of a clinical ED (Garner, 2004; Nyman-Carlsson et al., 2015). However, as differential associations of these ED symptoms with identity formation have not yet been thoroughly investigated, it is difficult to formulate clear hypotheses. In trying to do so, we refer to the transdiagnostic theory of EDs (Fairburn et al., 2003), which describes a dysfunctional self-evaluation as the core pathological process of all EDs. This dysfunctional self-evaluation comprises an overvaluation of shape and weight when evaluating the self, subsequently giving rise to body dissatisfaction in many of these individuals. Following this idea, body dissatisfaction would be closely associated with identity formation. Similarly, Kamps and Berman (2011) reported that distress related to identity confusion was a stronger predictor of body dissatisfaction than of overweight preoccupation in a community sample. Hence, we expected identity formation to show somewhat stronger temporal associations with body dissatisfaction, as compared with drive for thinness and bulimia symptoms.

Finally, the present study assessed whether these prospective identity-ED symptomatology associations would differ across gender and age groups, as both gender and age have been related to both constructs (Archer, 1989; Vandereycken and Noordenbos, 2008; Kroger et al., 2010; Opwis et al., 2017). With respect to gender, our analyses were of an explorative nature, as earlier research on this topic is rather scarce (Kerremans et al., 2010). With respect to age, we compared early adolescents (11–14 years) with mid- to late adolescents (15–19 years) at Time 1, as this categorization is often made in developmental psychology literature, especially when focusing on identity formation (Kroger, 2007). As previously described, identity confusion is often regarded a normative identity state in early adolescence that loses its functionality over time (Erikson, 1956). As identity maturation is expected throughout adolescence (Meeus et al., 2010), sustained identity confusion may give rise to feelings of insecurity and distress. Previous research has indeed pointed to stronger associations between identity confusion and depressive symptoms in emerging adulthood than in adolescence (Luyckx et al., 2013). Following this idea, we also hypothesize different identity-ED symptomatology associations throughout adolescence, with stronger associations in mid- to late adolescence than in early adolescence, assuming greater distress of experiencing identity confusion in this somewhat older age group.

## Materials and Methods

### Participants and Procedure

The present longitudinal study comprised three annual measurement waves (Time 1 being collected in January 2015, Time 2 in January 2016, and Time 3 in January 2017) as part of a larger data collection (Gandhi et al., 2017). Data were collected from high school students in Flanders, the Dutch speaking part of Belgium, through convenience sampling. Students that received consent from their parents were invited to participate in the study during school hours. They received an envelope holding an informed assent/consent form and the questionnaires. After participation, the envelope was returned to the researchers present at the school and was sealed. The same procedure was used at the second and third wave, with the added possibility for students that already left the school to fill in the questionnaire using an online web-survey to minimize drop-out. For each wave, participating students received a movie ticket as compensation. To ensure confidentiality, every student received a unique code number that was used during the entire study period. The study was approved by the ethics committee of the Faculty of Psychology and Educational Sciences, University of Leuven.

At Time 1, a total of 1,115 high school students were contacted for participation, of which 530 agreed (50.57% female; response rate = 47.53%). Mean age of participants at Time 1 was 15 years (*SD* = 1.85; range 11–19). At Time 2, 387 students agreed to participate (52.71% female, *M*_age_ = 15.52, *SD*_age_ = 1.68, age range 12–20; retention rate = 73.02%) and 327 students participated at Time 3 (54.63% female, *M*_age_ = 16.34, *SD*_age_ = 1.64, age range 13–21; retention rate = 61.70%). A total of 314 students participated at all three waves (59.25%). At both Times 2 and 3, drop-out was similarly associated with descriptive and study variables on the previous wave. Students that dropped out were somewhat older [Time 2: *M*_retention_ = 14.52 years (*SD* = 1.68), *M*_drop-out_ = 16.27 years (*SD* = 1.67), *F*(1,528) = 112.30, *p* < 0.001, η^2^ = 0.18; Time 3: *M*_retention_ = 15.28 years (*SD* = 1.60), *M*_drop-out_ = 16.52 years (*SD* = 1.64), *F*(1,383) = 34.99, *p* < 0.001, η^2^ = 0.08], were more likely to be female (Time 2: %female_retention_ = 53.23%, %female_drop-out_ = 43.36%, χ^2^(1) = 4.07, *p* = 0.044; Time 3: %female_retention_ = 55.10%, %female_drop-out_ = 42.47%, χ^2^(1) = 3.79, *p* = 0.052), but did not differ on adjusted BMI [Time 2: *F*(1,496) = 1.03, *p* = 0.310; Time 3: *F*(1,365) = 2.03, *p* = 0.156], identity formation [Time 2: *F*(2,523) = 0.77, *p* = 0.462; Time 3: *F*(2,383) = 0.20, *p* = 0.819], and ED symptomatology [Time 2: *F*(3,520) = 1.25, *p* = 0.292; Time 3: *F*(3,379) = 1.31, *p* = 0.272]. To compare students with and without complete data on the three waves, we conducted Little’s (1988) Missing Completely At Random (MCAR) test, which suggested that missing values were not associated with the observed data in the present study [χ^2^(121) = 129.14, *p* = 0.290]. However, as previously described, we did find dropout to be associated with gender and age, which contradicts these MCAR findings. Yet, the Full Information Maximum Likelihood (FIML) procedure was still used to handle missing values as it is typically less biased than *ad hoc* procedures such as listwise or pairwise deletion (Schafer and Graham, 2002).

### Measures

#### Adjusted Body Mass Index (BMI)

Students reported their weight and height, through which BMI (weight/height^2^) could be calculated. As earlier research suggests that self-report weight and height are validly reported in both adolescence and adulthood (Field et al., 1999), we did not assume a reporting bias in the present study. Additionally, as most students were underage, we computed the adjusted BMI [(BMI/Percentile 50 of BMI for age and gender) × 100] that takes into account the growth charts of a representative Flemish sample (Roelants and Hauspie, 2004) and allows determination of weight status (Van Winckel and Van Mil, 2001). At Time 1, 9.44% of the participating students were underweight (adjusted BMI ≤ 85), 82.33% had a normal weight (85 < adjusted BMI < 120), 6.83% were overweight (120 ≤ adjusted BMI < 140), and 1.41% were obese (140 ≤ adjusted BMI). This weight status-distribution is virtually equal to a study by Goossens et al. (2016) that also included male and female high-school students in Flanders.

#### Identity Formation

We used the Identity subscale of the Erikson Psychosocial Stage Inventory (EPSI; Rosenthal et al., 1981) that has good psychometric properties (Schwartz et al., 2009). The questionnaire exists of two subscales, each containing six items, scored on a 5-point Likert-type scale (from 1_*strongly disagree* to 5_*strongly agree*). Cronbach’s alphas for identity synthesis were 0.75, 0.74, and 0.79 at Times 1, 2, and 3, respectively. Cronbach’s alphas for identity confusion were 0.67, 0.70, and 0.74 at Times 1, 2, and 3, respectively.

#### Eating Disorder Symptomatology

The Eating Disorder Inventory-3 (EDI-3; Garner, 2004) is a valid questionnaire that taps into various ED symptoms and can be used as an ED screening tool in community samples (Lehmann et al., 2013; Nyman-Carlsson et al., 2015). The present study made use of the ED Risk Scales: drive for thinness, body dissatisfaction, and bulimia, adding up to 23 items scored on a 6-point Likert-type scale (untransformed scores ranging from 1_*never* to 6_*always*). Drive for thinness measures the desire to be thin and the general concern with dieting and body weight. Body dissatisfaction refers to the degree to which an individual is convinced that specific body parts (e.g., hips, thighs) are too large and is unsatisfied with their shape or form. Bulimia indicates the tendency to engage in binging and/or purging behaviors. At Times 1, 2, and 3, Cronbach’s alphas for drive for thinness were 0.91, 0.92, and 0.92, respectively, Cronbach’s alphas for body dissatisfaction were all 0.94, and Cronbach’s alphas for bulimia were 0.75, 0.79, 0.81, respectively.

### Statistical Analyses

For all preliminary analyses, we made use of IBM SPSS Version 24.0. First, repeated measures ANOVAs were used to examine mean changes for ED symptomatology and identity formation over the three waves. Next, to examine gender differences, we conducted a multivariate analysis of variance (MANOVA) with gender as fixed factor and ED symptomatology and identity formation at all time-points as dependent variables. Finally, Pearson correlations were calculated to investigate the associations among age and the study variables at all time-points.

To examine the directionality of effects between identity formation and ED symptomatology, we made use of cross-lagged path analysis from a structural equation modeling approach using Mplus version 7.4 (Muthén and Muthén, 2012). This technique allows for an indication of temporal precedence of variables that are longitudinally measured by estimating different types of relations among them: a *within-time association* refers to the relation between two variables measured at the same time, a *stability path* refers to the prediction of a variable at T2 by this same variable at T1, and a *cross-lagged path* refers to the prediction of a variable at T2 by another variable at T1 (Anderson and Kida, 1982; Kline, 2005).

To evaluate the model fit of the cross-lagged models, three fit-indices were used: the Satorra–Bentler scaled Chi-square (S–Bχ^2^), which uses a correction factor to account for non-normality and should be as small as possible in a larger sample size; the Comparative Fit Index (CFI), which should exceed 0.90 for reasonable fit and 0.95 for excellent fit; and the Root Mean Square Error of Approximation (RMSEA), which should be between 0.05 and 0.08 for reasonable fit and less than 0.05 for excellent fit (Satorra and Bentler, 2001; Kline, 2005). Two separate models were estimated, representing the temporal associations between (A) identity synthesis and ED symptomatology; and (B) identity confusion and ED symptomatology. The baseline models included all within-time associations and stability paths, and were both controlled for age and gender (which means that for each time point, paths from gender and age at baseline to the study variables were allowed). Further, all cross-lagged paths from identity formation to ED symptomatology and the cross-lagged paths in the opposite direction were included. In the next step, we constrained similar cross-lagged paths to be equal across time (e.g., the path from identity synthesis at T1 to drive for thinness at T2 would be constrained to be equal to the path from identity synthesis at T2 to drive for thinness at T3). To compare the model fit of the constrained model to the baseline model, we conducted a S–Bχ^2^ difference test (Satorra and Bentler, 2001).

Multi-group analyses were conducted to examine gender and age differences in the cross-lagged coefficients. Doing so, we compared a model in which the cross-lagged paths could vary across gender or age, respectively (i.e., unconstrained models), to a model in which the cross-lagged paths were constrained as equal across gender or age, respectively (i.e., constrained models). Finally, as an auxiliary analysis, we examined the cross-lagged models when additionally controlling for adjusted BMI at each time-point.

## Results

### Preliminary Analyses

Repeated measures ANOVAs pointed to non-significant mean changes for drive for thinness [Wilks’Λ = 1.00, *F*(2,305) = 0.02, *p* = 0.985] and body dissatisfaction [Wilks’Λ = 1.00, *F*(2,304) = 0.21, *p* = 0.808] over the 2-year period. Bulimia symptoms significantly increased over the 2-year period [Wilks’Λ = 0.96, *F*(2,305) = 6.79, *p* = 0.001], as students reported significantly more bulimia symptoms at Time 3 (*M* = 2.00, *SD* = 0.82) than at Times 1 (*M* = 1.87, *SD* = 0.77) and 2 (*M* = 1.89, *SD* = 0.76). Mean levels of EDI subscales were similar to findings from previous studies focusing on ED symptomatology in a community sample and were substantially lower than in studies focusing on clinical (ED) samples (Lehmann et al., 2013; Nyman-Carlsson et al., 2015)^1^. Identity formation remained stable over time [synthesis: Wilks’Λ = 1.00, *F*(2,307) = 0.29, *p* = 0.748; confusion: Wilks’Λ = 1.00, *F*(2,309) = 0.15, *p* = 0.858]. With regard to gender differences, a significant effect was found [Wilks’Λ = 0.73, *F*(15,263) = 6.46, *p* < 0.001]. **Table 1** displays follow-up univariate *F*-values, indicating that women tend to score higher on ED symptomatology (DT, B, BD), and lower on identity synthesis than men. Regarding age, correlational analyses only indicated significant associations at Time 1, with age being negatively associated with identity synthesis (*r* = -0.14, *p* < 0.05) and positively associated with identity confusion (*r* = 0.14, *p* < 0.05), body dissatisfaction (*r* = 0.13, *p* < 0.05), and bulimia symptoms (*r* = 0.14, *p* < 0.05). Finally, **Table 2** presents the correlations among all study variables. ED symptomatology was negatively related to identity synthesis and positively related to identity confusion at all three time-points.

**Table 1:** Gender distribution of ED symptomatology and identity formation with means, standard deviations, and univariate ANOVAs.

Variables	Time 1			Time 2			Time 3		
	Males *M* (*SD*)	Females *M* (*SD*)	*F*(1,303)	Males *M* (*SD*)	Females *M* (*SD*)	*F*(1,303)	Males *M* (*SD*)	Females *M* (*SD*)	*F*(1,303)
**ED symptomatology**									
Drive for thinness	1.85 (0.92)	2.76 (1.28)	48.14^∗∗∗^	1.80 (0.81)	2.79 (1.31)	60.15^∗∗∗^	1.78 (0.86)	2.82 (1.30)	63.90^∗∗∗^
Body dissatisfaction	2.24 (0.99)	3.44 (1.27)	80.40^∗∗∗^	2.29 (0.96)	3.43 (1.21)	79.26^∗∗∗^	2.29 (1.03)	3.45 (1.23)	77.82^∗∗∗^
Bulimia	1.81 (0.62)	1.91 (0.88)	1.47	1.79 (0.63)	1.97 (0.85)	3.91^∗^	1.86 (0.69)	2.12 (0.90)	7.85^∗∗^
**Identity formation**									
Identity synthesis	3.78 (0.58)	3.63 (0.58)	4.94^∗^	3.81 (0.56)	3.61 (0.57)	8.92^∗∗^	3.79 (0.60)	3.59 (0.68)	7.50^∗∗^
Identity confusion	2.54 (0.61)	2.68 (0.62)	3.90^∗^	2.52 (0.60)	2.66 (0.66)	4.16^∗^	2.50 (0.61)	2.70 (0.69)	7.11^∗∗^

*^∗^p < 0.05, ^∗∗^p < 0.01, ^∗∗∗^p < 0.001.*

**Table 2:** Correlations among the study variables.

Variables	2	3	4	5	6	7	8	9	10	11	12	13	14	15
**ED symptomatology**		
(1) DT T1	0.80^∗∗^	0.69^∗∗^	0.78^∗∗^	0.72^∗∗^	0.64^∗∗^	0.42^∗∗^	0.35^∗∗^	0.37^∗∗^	–0.27^∗∗^	–0.32^∗∗^	–0.24^∗∗^	0.33^∗∗^	0.28^∗∗^	0.27^∗∗^
(2) DT T2		0.78^∗∗^	0.69^∗∗^	0.79^∗∗^	0.69^∗∗^	0.38^∗∗^	0.43^∗∗^	0.39^∗∗^	–0.25^∗∗^	–0.33^∗∗^	–0.27^∗∗^	0.28^∗∗^	0.31^∗∗^	0.28^∗∗^
(3) DT T3			0.60^∗∗^	0.70^∗∗^	0.79^∗∗^	0.28^∗∗^	0.34^∗∗^	0.43^∗∗^	–0.19^∗∗^	–0.30^∗∗^	–0.29^∗∗^	0.22^∗∗^	0.25^∗∗^	0.33^∗∗^
(4) BD T1				0.83^∗∗^	0.73^∗∗^	0.40^∗∗^	0.32^∗∗^	0.34^∗∗^	–0.35^∗∗^	–0.37^∗∗^	–0.32^∗∗^	0.42^∗∗^	0.34^∗∗^	0.34^∗∗^
(5) BD T2					0.84^∗∗^	0.34^∗∗^	0.36^∗∗^	0.38^∗∗^	–0.32^∗∗^	–0.43^∗∗^	–0.40^∗∗^	0.36^∗∗^	0.38^∗∗^	0.40^∗∗^
(6) BD T3						0.34^∗∗^	0.38^∗∗^	0.47^∗∗^	–0.26^∗∗^	–0.37^∗∗^	–0.40^∗∗^	0.32^∗∗^	0.34^∗∗^	0.42^∗∗^
(7) B T1							0.68^∗∗^	0.59^∗∗^	–0.34^∗∗^	–0.34^∗∗^	–0.32^∗∗^	0.45^∗∗^	0.33^∗∗^	0.36^∗∗^
(8) B T2								0.74^∗∗^	–0.30^∗∗^	–0.33^∗∗^	–0.29^∗∗^	0.33^∗∗^	0.32^∗∗^	0.36^∗∗^
(9) B T3									–0.23^∗∗^	–0.36^∗∗^	–0.31^∗∗^	0.28^∗∗^	0.33^∗∗^	0.39^∗∗^
**Identity formation**		
(10) IS T1										0.58^∗∗^	0.48^∗∗^	–0.64^∗∗^	–0.43^∗∗^	–0.42^∗∗^
(11) IS T2											0.68^∗∗^	–0.49^∗∗^	–0.66^∗∗^	–0.60^∗∗^
(12) IS T3												–0.42^∗∗^	–0.48^∗∗^	–0.68^∗∗^
(13) IC T1													0.59^∗∗^	0.56^∗∗^
(14) IC T2														0.63^∗∗^
(15) IC T3														–

*DT, drive for thinness; BD, body dissatisfaction; B, bulimia; IS, identity synthesis; IC, identity confusion; ^∗∗^p < 0.01.*

### Directionality of Effects Linking Identity Formation and ED Symptomatology

**FIGURE 1:**
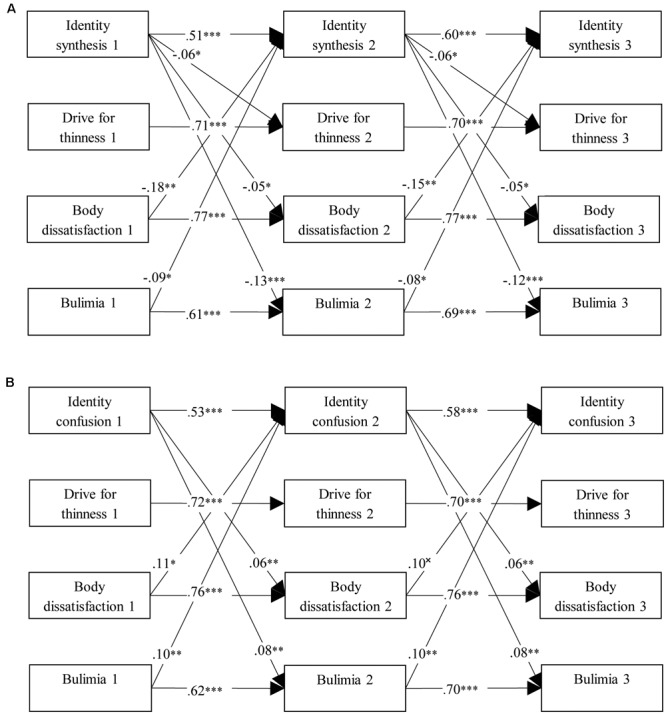
Final cross-lagged models linking ED symptomatology to **(A)** identity synthesis and **(B)** identify confusion. Both models were controlled for age and gender. Within-time associations, associations with age and gender, and insignificant cross-lagged paths were not shown for reasons of clarity. All path coefficients are standardized. ^×^*p* = 0.05, ^∗^*p* < 0.05, ^∗∗^*p* < 0.01, ^∗∗∗^*p* < 0.001.

Cross-lagged path analyses resulted in baseline models with acceptable fit [Model A: S–Bχ^2^(28) = 92.31, *p* < 0.001; CFI = 0.98; RMSEA = 0.07; and Model B: S–Bχ^2^(28) = 108.47, *p* < 0.001; CFI = 0.98; RMSEA = 0.07]. In addition, model comparison indicated that the constraints on the cross-lagged paths were allowed for both models [Model A: ΔS–Bχ^2^(6) = 3.62, *p* = 0.728; and Model B: ΔS–Bχ^2^(6) = 9.85, *p* = 0.127], which indicates that the cross-lagged paths could be constrained equal across time. The two final models are presented in **Figure 1**. In the first cross-lagged model (Model A, **Figure 1A**), identity synthesis negatively predicted all ED symptoms over time, whereas body dissatisfaction and bulimia symptoms negatively predicted identity synthesis over time. When looking at Model B (**Figure 1B**), identity confusion positively predicted body dissatisfaction and bulimia symptoms over time, and vice versa.

Multi-group analyses for gender and age indicated that the cross-lagged models in which paths were constrained as equal across gender/age did not have a worse fit to the data: the cross-lagged models fitted equally well for males and females [Model A: ΔS–Bχ^2^(6) = 5.90, *p* = 0.434; and Model B: ΔS–Bχ^2^(6) = 3.29, *p* = 0.771], as well as for early adolescents and mid- to late adolescents [Model A: ΔS–Bχ^2^(6) = 11.96, *p* = 0.063 and Model B: ΔS–Bχ^2^(6) = 5.45, *p* = 0.488]. In sum, cross-lagged paths were not moderated by age or gender.

### Auxiliary Analyses

The final cross-lagged models described above where controlled for both gender and age. When additionally controlling for adjusted BMI at each time-point, the same cross-lagged paths emerged as in the original model with regard to the associations between identity confusion and ED symptomatology (Model B). When looking at the associations between identity synthesis and ED symptomatology (Model A), almost all cross-lagged paths emerged as in the original model, except for the cross-lagged path from bulimia symptoms to identity synthesis, which was not significant when controlled for adjusted BMI (*p*_1_ = 0.055 and *p*_2_ = 0.054). Overall, additionally controlling for adjusted BMI did not seem to significantly change the estimates for the cross-lagged paths between identity formation and ED symptomatology.

## Discussion

Disturbed eating behaviors and body image problems are important issues in adolescent boys and girls (Ricciardelli and McCabe, 2001; Croll et al., 2002). The present study aimed to enhance our understanding of the emergence and development of ED symptoms in adolescence by focusing on identity formation, a key developmental task in this life period. The three-wave longitudinal design of the present study offered valuable insights into the directionality of effects linking identity formation to ED symptomatology in a community sample of adolescents. Results indicated a bidirectional relation between identity development and ED symptomatology over time. More specifically, identity synthesis seemed to protect against all ED symptoms over time, whereas body dissatisfaction and bulimia seemed to hinder the development of identity synthesis over time. Finally, bidirectional pathways were found between identity confusion on the one hand and body dissatisfaction and bulimia symptoms on the other hand. Identity confusion seemed to positively predict these symptoms over time, whereas these symptoms also positively predicted identity confusion.

With regard to the longitudinal study design, it is important to notice that students that dropped out during the study seemed somewhat older and less likely to be female than students that participated at all waves. As girls are generally found to be more conscientious than boys (Vecchione et al., 2012), it is not surprising that they tend to show a smaller drop-out percentage in the present study. Additionally, the age difference between the drop-out group and the retention group was anticipated, as a larger drop-out rate was expected for students that had left high school at the second or third wave and had to be contacted individually via e-mail.

Across the studied 2-year period, both identity synthesis and confusion remained stable, which indicates that the study sample presented with the same mean scores on identity functioning each year, pointing to the rather slow and gradual process of identity maturation (Marcia, 1980; Waterman, 1993). A review of empirical studies on identity development (Meeus et al., 1999) indeed found that, in many of the studies reviewed, an overall stability in identity was found in high school students. Similarly, drive for thinness and body dissatisfaction remained stable over time, supporting earlier research on the stability of dieting behavior throughout adolescence for both boys and girls (Neumark-Sztainer et al., 2011). Bulimia symptoms did somewhat increase with age, with a significant difference between the last and first two time-points. Hence, although adolescents experienced consistent levels of body dissatisfaction and the desire to be thinner over time, they did seem to engage more in impulsive and compensatory eating behavior such as binge eating and purging. These findings support previous research that found an increase in bulimic symptoms throughout mid- to late adolescence in high school girls and described late adolescence as the most at-risk age group for developing bulimia nervosa (Stice et al., 2009; Abebe et al., 2012).

With regard to gender differences, our findings were consistent with previous studies indicating that women generally experience greater ED symptomatology than men (Lewinsohn et al., 2002). These results confirm that even in adolescence, women tend to be less satisfied with their body shape and engage more in disturbed eating behavior when compared to men. This finding further supports the idea that especially women may be vulnerable to the thin ideal of western society, that often drives them to idealize and strive for a slim and slender body (Lawler and Nixon, 2011). With respect to identity, women scored somewhat lower on identity synthesis than men. This is partially in line with earlier research describing (1) women generally needing more time before adhering to a set of self-identified choices (Luyckx et al., 2008; Verschueren et al., 2017b) and (2) men scoring higher on decision making and identity commitments than women (Archer, 1989; Verschueren et al., 2017b). These results could help in explaining the gender differences on identity synthesis in the present study.

With respect to our primary objective, the obtained findings were in line with literature reporting a relationship between identity formation and ED symptomatology. Correlational analyses indicated a negative association between ED symptoms and identity synthesis and a positive association between ED symptoms and identity confusion. However, the main purpose of this study was to determine the directionality of effects linking both constructs. Cross-lagged path analysis indicated that identity formation and ED symptomatology seemed to predict one another over time throughout adolescence. As expected (Casper, 1983; Wheeler et al., 2001; Kamps and Berman, 2011), both identity synthesis and identity confusion were related to ED symptoms over time, with identity confusion possibly increasing one’s vulnerability to the development of ED symptoms and identity synthesis protecting against these symptoms. Adolescents with greater doubts about certain identity choices may be more susceptible to socially excepted goals and values and tend to conform more to expectations of others, instead of taking a critical stance toward identity issues (Berzonsky, 2004). Consequently, these adolescents are more vulnerable to adopt the perfect body ideal of western society (Verstuyf et al., 2014) and experience greater dissatisfaction about their body shape and size. Indeed, the close relation between identity and body dissatisfaction was present in both of our cross-lagged models. In line with expectations, identity problems seemed to increase vulnerability to bulimia symptoms over time as well. As previous research has described, binging and purging behaviors are considered (maladaptive) emotion regulation strategies in certain individuals (Stice et al., 1996; Lavender et al., 2014). Hence, as identity confusion is often accompanied by great distress (Erikson, 1968), binging and purging may function as maladaptive strategies to regulate, avoid, or escape negative affect that is associated with identity distress (Heatherton and Baumeister, 1991; Wheeler et al., 2001).

Although most theories and studies focus on the idea that identity problems underlie ED symptoms, the present study found evidence for the reverse pathways as well, in which ED symptoms seemed to hamper identity development over time. As previously described, individuals with disturbed eating behavior generally experience an overvaluation of body size and weight, causing a narrowly defined sense of identity (Stice, 2002; Fairburn et al., 2003; Corning and Heibel, 2016). In this case, the body represents a core identity aspect and although this focus may offer clarity and empowerment to some (i.e., being some sort of pseudo-identity; Nordbø et al., 2006; Rich, 2006), alternate highly valued self-aspects cannot develop properly. Consequently, body dissatisfaction would be experienced as a direct threat for one’s identity, as other sources of self-esteem are lacking (Corning and Heibel, 2016). The present findings indeed point to body dissatisfaction hampering identity synthesis and increasing identity confusion over time. Additionally, results showed the same pattern for identity and bulimia symptoms, in which bulimia symptoms seem to hamper identity development. Again, the emotional regulatory capacities of bulimia symptoms could offer a theoretical framework to understand this effect. As binging and purging may act as an avoidance or escape mechanism for certain individuals to deal with identity issues (Stice et al., 1996; Lavender et al., 2014), engaging in these types of behaviors could seriously hamper constructive identity work.

Surprisingly, looking at the cross-lagged models for both identity synthesis and identity confusion, we did not find consistent pathways for drive for thinness, as it was only (negatively) related to identity synthesis over time – identity synthesis seemed to protect against drive for thinness – but was not related to identity confusion. In interpreting this result, several authors already described the ambiguous nature of drive for thinness with respect to identity development, underscoring that the construct does not always hold a clear negative association with it. Bruch (1981) described how focusing one’s attention on body weight may offer a viable source of self-definition to some individuals, and Casper (1983) pointed out that in adolescence and young adulthood, striving for a thin body may sometimes be related to a firmer sense of identity. Future research may wish to focus on different goal pursuits possibly underlying a drive for thinness. More specifically, inspired by the Self-Determination Theory (Deci and Ryan, 2008), whereas some individuals may strive for thinness mainly for health concerns, other individuals may hope to lose weight to attain certain beauty ideals. As Luyckx et al. (2017) found that certain identity processes may be differentially related to such intrinsic versus extrinsic goal pursuit (Soenens and Vansteenkiste, 2011), it could be possible that one’s drive for thinness would be related to both identity synthesis and confusion, albeit through different types of goal pursuit. However, these interpretations should be handled with caution as the present findings cannot fully confirm them.

Finally, with regard to gender and age differences, the present study found no significant differences in the identity-eating disturbance interplay between adolescent women and men, neither between early adolescents and mid- to late adolescents. These findings are interesting and clinically relevant as men are often overlooked in ED research. The present study suggests that, although men generally seem to experience less ED symptoms than women, those that do experience body dissatisfaction or engage in bulimia symptoms, seem to be at risk for experiencing identity confusion, and vice versa, much in the same vein as women do.

Although the study offers new insights in the identity-ED link, several limitations need to be considered. First, the study was carried out in a western culture and made use of convenience sampling. As eating concerns and the body perfect ideal are culturally specific (Warren et al., 2005; Townsend et al., 2014), our findings may not be transferable to non-western cultures. Cross-cultural studies are needed to compare the identity-ED symptoms interplay across different cultures. Additionally, due to the sampling technique, selection bias cannot be excluded. It could be possible that adolescents experiencing serious ED-symptoms chose not to participate to the study to avoid possible negative affect due to filling out the questionnaires. Representative samples are recommended in future research. Second, the present study made exclusive use of self-report questionnaires. Although identity formation and most ED symptoms are internal and subjective processes, the inclusion of other methods (e.g., interview) and other-reported measures may be helpful to confirm our results. Third, the study did not include measures such as excessive exercise, which have been described as prominent ED problems for men especially (Touyz et al., 1993; Micali et al., 2014). As the body perfect ideal generally depicts a strong and muscular body for men, men who experience more identity confusion, may not only score higher on body dissatisfaction, but also on excessive exercise. Hence, including this measure in future research could offer further insights on this matter. Finally, the present study made use of Erikson’s identity model, focusing on identity synthesis versus identity confusion. New identity models have been developed that offer a more dynamic look on identity (Crocetti et al., 2008; Luyckx et al., 2008). It would be interesting to look at the temporal sequence of identity formation and ED symptomatology, using these more dynamic models on identity as well.

Despite these limitations, the present three-wave study adds to the existing literature, as it was the first to investigate the temporal sequence of identity and ED symptomatology from early to late adolescence. The findings support the tenet that identity confusion increases the development of ED symptoms in both adolescent women and men, but – contrary to most proposed theories – ED symptoms seem to hamper the identity development as well. This developmental interplay should be taken into account when developing ED prevention programs. Bolstering one’s identity may avoid the development of body dissatisfaction and bulimia symptoms in the future, and similarly, focusing on a healthy body image may prevent serious identity issues (Corning and Heibel, 2016). Additionally, ED intervention programs should aim to break the vicious circle of identity confusion and ED symptomatology in both adolescent women and men. It seems important to identify patients that experience higher levels of identity confusion, as therapists could help them develop a stronger identity, which could reduce general ED symptomatology.

## Ethics Statement

This study was carried out in accordance with the recommendations of the ethics committee of the Faculty of Psychology and Educational Sciences, University of Leuven, which approved the protocol. All subjects gave written informed consent in accordance with the Declaration of Helsinki.

## Author Contributions

AG and LC oversaw the data collection. MV, KL, and LC conceived of the presented idea, constructed the hypotheses, analyzed the data, and interpreted the results. MV wrote the manuscript with critical revision from all authors (KL, LC, AB, NP, AG, and PM).

## Conflict of Interest Statement

The authors declare that the research was conducted in the absence of any commercial or financial relationships that could be construed as a potential conflict of interest. The handling Editor declared a past co-authorship with one of the authors LC.

## Footnotes

1 The present study made use of untransformed item scores (ranging from 1 to 6), with DT, BD, and B representing mean scale scores. Comparing mean levels on all time points, DT was 2.35 at all time points, BD ranged from 2.90 to 2.94, and B ranged from 1.87 to 2.00. To compare these mean levels to other studies using the transformed item scores of the EDI-3, we transformed the item scores to a 5-point format (0, 0, 1, 2, 3, 4), after which we calculated DT, BD, and B as sum scale scores. Mean levels on these sum scores ranged from 5.25 to 6.01 for DT, from 10.83 to 11.42 for BD, and from 2.97 to 3.55 for B.
